# Microglial Function during Glucose Deprivation: Inflammatory and Neuropsychiatric Implications

**DOI:** 10.1007/s12035-017-0422-9

**Published:** 2017-02-07

**Authors:** Matthew A. Churchward, Devan R. Tchir, Kathryn G. Todd

**Affiliations:** 1 Neurochemical Research Unit, Department of Psychiatry, University of Alberta, 116th St and 85th Ave NW, Edmonton, AB T6G2R3 Canada; 2 Neuroscience and Mental Health Institute, University of Alberta, Edmonton, AB T6G 2R3 Canada

**Keywords:** Microglia, Ischemia, Inflammation, Depression, Hypoglycemia, Diabetes

## Abstract

Inflammation is increasingly recognized as a contributor to the pathophysiology of neuropsychiatric disorders, including depression, anxiety disorders and autism, though the factors leading to contextually inappropriate or sustained inflammation in pathological conditions are yet to be elucidated. Microglia, as the key mediators of inflammation in the CNS, serve as likely candidates in initiating pathological inflammation and as an ideal point of therapeutic intervention. Glucose deprivation, as a component of the pathophysiology of ischemia or occurring transiently in diabetes, may serve to modify microglial function contributing to inflammatory injury. To this end, primary microglia were cultured from postnatal rat brain and subject to glucose deprivation in vitro*.* Microglia were characterized for their proliferation, phagocytic function and secretion of inflammatory factors, and tested for their capacity to respond to a potent inflammatory stimulus. In the absence of glucose, microglia remained capable of proliferation, phagocytosis and inflammatory activation and showed increased release of inflammatory factors after presentation of an inflammatory stimulus. Glucose-deprived microglia demonstrated increased phagocytic activity and decreased accumulation of lipids in lipid droplets over a 48-h timecourse, suggesting they may use scavenged lipids as a key alternate energy source during metabolic stress. In the present manuscript, we present novel findings that glucose deprivation may sensitize microglial release of inflammatory mediators and prime microglial functions for both survival and inflammatory roles, which may contribute to psychiatric comorbidities of ischemia, diabetes and/or metabolic disorder.

**Electronic supplementary material:**

The online version of this article (doi:10.1007/s12035-017-0422-9) contains supplementary material, which is available to authorized users.

## Introduction

Inflammation of the central nervous system, regulated by microglia—the resident immune cells, is an acute reaction to the presence of infectious agents, foreign bodies or tissue damage intended to protect, contain and repair vulnerable CNS tissues. In an increasing number of neuropathologies, prolonged, inappropriate or chronic inflammation has been shown to exacerbate damage to surrounding neural and glial cells. Inflammation is implicated in the development of numerous neurodegenerative disorders, including Alzheimer’s [1, 2], Parkinson’s [3, 4] and Huntington’s diseases [5], acute conditions including ischemic and hemorrhagic stroke [6–9], traumatic brain injury [10–12] and spinal cord injury [13] and neuropsychiatric disorders including depression and anxiety disorders [14–16], schizophrenia [17–20], autism [21–24] and post-traumatic stress disorder [25, 26]. While recent studies have shed light on both the importance of microglia-mediated inflammation in normal repair processes and the evidence for pathological inflammation in neuropsychiatric disorders, much remains unknown about the mechanisms and triggers involved in normal and pathological inflammation, and the divergent functions of microglia in carrying out both trophic and toxic roles in the CNS.

Inflammation as a component of psychiatric disorders has a number of possible sources. A recently popularized hypothesis postulates the aetiology of depression may derive, in part, from prolonged or chronic immune activation [15, 27, 28]. Consistent with this hypothesis, a number of studies have found elevated peripheral cytokines (particularly tumour necrosis factor (TNF) and interleukin-6 (IL-6)) in depressed patients, and depression is the most observed psychiatric comorbidity of medical illness [29]. Rates of depression are elevated in a wide range of medical conditions including coronary heart disease, cancer, diabetes, ischemic stroke and in particular chronic inflammatory diseases (autoimmune disorders, rheumatoid arthritis, inflammatory bowel disease, asthma and allergies) [28, 30–34]. While many have argued that comorbid depression is expected as a consequence of ‘feeling bad’ about having a chronic illness, comorbid depression paradoxically does not necessarily correlate with disease prognosis (e.g. in cancers with positive expected outcome) nor with disease severity (e.g. in the case of asthma and allergies, which are both manageable and can have limited impact on daily life) [27]. An alternate explanation of comorbid depression is that clinical depression is difficult to distinguish from *sickness behaviours*, i.e. depressed mood, anhedonia, social withdrawal, appetite changes and fatigue as an adaptive phenotype to prioritize recovery from infection or illness [35, 36]. Supporting this argument is the observation that sterile inflammation, through administration of either cytokines (interferon-α (IFN-α) or IL-2 as immune boosters for hepatitis C or cancer treatments) or bacterial lipopolysaccharide (LPS), results in manifestation of depressive symptoms [37–39]. By extension, observed increases in inflammatory markers in otherwise medically healthy depressed patients suggests depression, in some cases, may result from maladaptive expression of sickness behaviours in the absence of infection or illness. Such observations in psychiatric and neurological disease has led to recent understanding that inflammation is a key component of the pathophysiology of many neurological, neurodegenerative and neuropsychiatric disorders, and is encouraging research into the mechanisms underlying inflammatory processes in the CNS [18].

The desire to understand inflammatory processes in the CNS necessitates an understanding of microglia. Microglia are functionally unique in the CNS, and are distinguished in part by their persistent function in environments that are adverse to the survival of neurons and other glial cells. This includes relatively minor disruptions of homeostasis, such as alterations in extracellular pH and nutrient levels, to extremely adverse environments such as the core of an ischemic injury [40]. Such an environment is devoid of blood flow, and hence subject to deprivation of oxygen, glucose and other nutrients, accompanied by loss of neurons, astrocytes, oligodendrocytes and other resident cells, yet microglia survive, proliferate and migrate to the ischemic infarct [41–44]. Inflammation as a consequence of ischemia has been best studies in models of ischemic stroke, though may significantly contribute to the pathogenesis of neurodegenerative and neuropsychiatric disease. Depression is the most common psychiatric comorbidity following ischemic stroke, and has a significant effect on recovery and prognosis [45], while some have proposed that depression, particularly in ageing patients, may result from subcortical ischemia due to vascular insufficiency [46, 47]. In a similar fashion, age-related cognitive impairment and dementia is a well-documented consequence of mild or chronic ischemia [48–51], and some have proposed dementia associated with Alzheimer’s may result in part from local hypoxic/ischemic conditions [52–54]. Transient glucose deprivation in the absence of hypoxia can occur in diabetes, particularly in patients with insulin resistance, and is considered a major clinical concern in diabetes management [55]. Type II diabetes has a well-characterized bidirectional association with depression: patients with type II diabetes are at an increased risk of depression, and patients with depression are at an increased risk of developing type II diabetes (reviewed by Berge and Riise [56]). Several mechanisms have been proposed to explain the relationship between depression and diabetes including the influence of shared lifestyle and socioeconomic risk factors, converging effects of cytokines and cortisol on both insulin resistance and CNS inflammation and the contribution of advanced glycation end-products to inflammatory processes and subsequent effects on cognitive function [56–59]. Imaging work has demonstrated a relationship between a history of severe hypoglycemic events and decreased grey matter density in patients with type I diabetes [60], raising the intriguing possibility that variation in glucose levels, persistent hyperglycemia and transient hypoglycemia, may each contribute to neuropathology, cognitive impairment and psychiatric comorbidities in diabetes. Consequently, an improved understanding of the effects of glucose deprivation on the function of microglia will provide insight into mechanisms underlying neuroinflammation as relates to neuropsychiatric and neurodegenerative disease, particularly comorbid depression and diabetes.

Much of our understanding of the role of microglia in inflammation comes by analogy to the role of macrophages in peripheral inflammation. Indeed, given the relative challenge and expense of either fresh isolating or culturing primary microglia from human tissues, isolation of monocytes and their polarization into macrophages has remained one of the few viable reductionist systems for mechanistic studies of inflammation in human cells. Animal studies have demonstrated remarkable differences exist between the form and function of macrophages and microglia, particularly as relates to CNS homeostasis and repair. While both cell types can be involved in CNS inflammation and restoration of homeostasis, they behave very differently [61]. Microglia respond to a variety of factors by taking on a pro-inflammatory state that both morphologically and functionally resembles a macrophage, engaging in phagocytosis of cellular debris and secretion of pro-inflammatory factors such as cytokines (IL6, IL1β, and TNF), chemokines (chemokine (C-C motif) ligand 3, 5 (CCL3, CCL5)) and reactive oxygen/nitrogen species (nitric oxide, peroxides, superoxide, peroxynitrite) to regulate the fate of pathogens and vulnerable neurons; however, aside from this specific state, the similarities are greatly diminished [62–65]. Microglial *activation* is a misnomer that is unfortunately widely propagated in the literature—microglia are constitutively active across a continuum of states leading to expression of a wide variety of cellular behaviours [66, 67]. In the surveillant or homeostatic state in healthy CNS tissue, microglia are highly active, extending and modifying their branched arborizations to physically sample the entire volume of the CNS approximately every 2 h [68–71]. These microglia are territorial: two adjacent microglia will maintain respectful boundaries in order to prevent redundant oversampling of the parenchyma. In response to internal or external stimuli, microglia enter a reactive state and are capable of secreting a wide variety of substances to modify the function of surrounding cells. This includes factors such as are described for ‘classical’ and ‘alternate’ activation of macrophages to the M1 and M2 phenotypes, respectively; however, these are far from discrete states in microglial populations—these represent a snapshot of activity along a functional continuum, and in fact microglia have been demonstrated to simultaneously express both M1 and M2 markers [67, 72]. In further contrast to macrophages—thought to polarize towards either secretory or phagocytic phenotypes, microglia are highly capable of phagocytosis across their functional continuum, including specific developmental and plasticity-related roles in activity-dependent synaptic pruning and phagocytic regulation of neuronal precursor cell populations which occur in the absence of overt inflammatory conditions [73–75]. Microglia and macrophages vary in their response to CNS injury—recent work has shown that macrophages, whether polarized to the inflammatory M1 phenotype or the ‘trophic’ M2 phenotype, are injurious to neurons in an ex vivo model of ischemic injury, while microglia are uniquely capable of protecting and promoting recovery of vulnerable neurons [76]. Further, as a resident population, microglia are self-sustaining: both microglia and macrophages proliferate in response to inflammatory stimuli; however, macrophages have a terminal endpoint after resolution of inflammation while microglia are capable of returning to a surveillant state to restore the homeostatic population [77, 78]. These studies demonstrate the need for direct investigations into the specific roles of microglia in the initiation, propagation and resolution of inflammation in the CNS.

Ischemia is generally modelled in vitro using oxygen and glucose deprivation (OGD), mimicking two of the key insults to occur during ischemia. In either ex vivo slice culture preparations or isolated in vitro cultures, microglia respond to OGD by increasing secretion of both pro-inflammatory cytokines and trophic growth factors [76, 79–81]. This release highlights one of the therapeutic challenges of targeting inflammation: microglia are both essential for repair and to limit spread of ischemic injury, and contributors to secondary damage in ischemic tissue [82–84]. The effects of hypoxia on microglia are well documented: activation of hypoxia-inducible factor-1α (HIF1α) leads to transcription of numerous genes, including expression of pro-inflammatory cytokines [85–87]. HIF-1α is also responsible for alteration of glycolytic pathways to favour anaerobic metabolism [88] to prevent energy failure and promote cell survival. Recent work has suggested that hypoxia and glucose deprivation may have antagonistic effects on release of inflammatory cytokines [89], which may also contribute to conflicting reports in the literature regarding the survival of microglia after OGD [80, 90, 91].

Interestingly, very few reports have sought to determine the direct effects of glucose deprivation (GD) on microglial function. In a recent study, Choi and colleagues reported that glucose deprivation increased gene expression and secretion of the cytokine Il-6 from both immortalized microglial cell lines and primary murine microglia in vitro, and these changes were antagonized by coincident application of hypoxia [89]. While these specific findings suggest GD, but not hypoxia, increased inflammatory release, it is important to note that effects on a single cytokine cannot be generalized to an overall effect on release of inflammatory mediators, particularly as hypoxia is a well-identified activator of inflammation both in vitro and in vivo [85–87]. Given the correlation between ischemia/hypoglycemia and psychiatric disorders (notably depression) and lack of available evidence, we proposed the following question: *what effect does the availability of glucose have on the initiation of inflammation in the CNS?* As pro-inflammatory functions of microglia are energy intensive, requiring new protein synthesis and extensive cytoskeletal reorganization, it is perhaps unsurprising that treatment with inflammatory stimuli such as LPS and IFN-γ results in increased glucose uptake and utilization [92, 93] and result in distinct metabolic changes to support such vital functions [94]. Given these energy demands, and previous work suggesting antagonistic effects of hypoxia and hypoglycemia, we proposed the following specific hypothesis: glucose deprivation will render microglia less capable of releasing inflammatory modulators when presented with an exogenous inflammatory stimulus. As an initial test of this hypothesis, we determined the effects of GD on primary cultured microglia, and found that contrary to our hypothesis glucose-deprived microglia showed comparable or increased capacity for phagocytic and inflammatory function.

## Methods

### Materials

DMEM/Ham’s F-12 (DMEM/F-12, 1:1), foetal bovine serum (FBS), penicillin/streptomycin, 0.25% trypsin/EDTA and Hank’s balanced saline solution (HBSS) were from Gibco (Thermo-Fisher Scientific, Ottawa, ON). Bodipy 493/503 and Click-IT EdU Imaging kit were from Molecular Probes (Thermo-Fisher Scientific, Ottawa, ON). ELISA kits for IL1β and TNF were from R&D Systems (Minneapolis, MN, USA). All other reagents were of the highest quality available.

### Primary Microglial Cell Culture and Treatments

Microglia were isolated from mixed glial cultures at 14 days in vitro (d.i.v.) according to the method of Saura [95]. Whole brains were isolated from male postnatal day 1 rat pups, meninges removed and single cell suspensions generated by trypsinization and trituration. Mixed glial cultures were maintained on 12-well plates with DMEM/F-12, 10% FBS, 200 U/ml penicillin, 200 μg/ml streptomycin in a 37 °C, 5% CO_2_ humidified incubator for 14 days and isolated by mild trypsinization (0.25% trypsin/EDTA diluted to 30% strength with DMEM/F12). Isolated cultures were consistently of ≥98% purity in all conditions and experiments, and were maintained in DMEM/F12 with 1% FBS for 18 h prior to start of experiments. All animal procedures were performed in accordance with the University of Alberta animal care and use committee’s regulations.

**Fig. 1:**
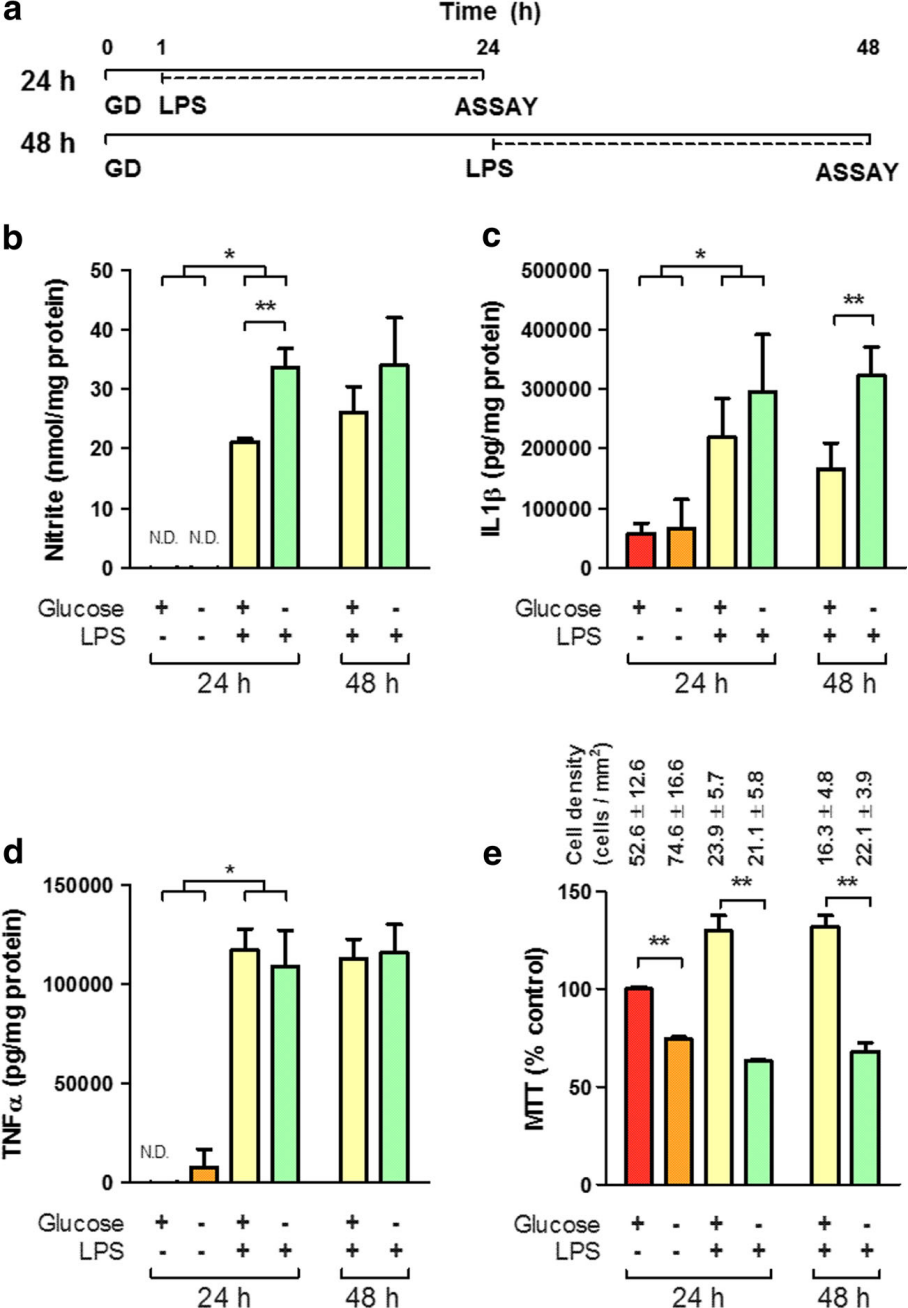
Glucose deprivation is insufficient to induce inflammatory release from microglia. **a** Schematic timeline of treatment paradigm. For each condition, GD began 1 or 24 h (for 24 or 48 h total time points, respectively) prior to 24 h 100 ng/ml LPS treatment. **b**–**d** Release of NO (**b**), IL1β (**c**) and TNF (**d**) is significantly increased by treatment with LPS. GD increases release of NO and IL1β significantly above the LPS treated, normal glucose condition at 24 and 48 h, respectively (*N* = 4). MTT assay (**e**), a measure of oxidative phosphorylation, was significantly decreased in microglia subject to glucose deprivation relative to normal glucose at 24 h, and in the presence of LPS at 24 or 48 h (*N* = 8). *Numbers* indicate mean cell density for each condition as determined from a parallel assay plate. *N.D.* not determinable. *Asterisk* indicates significant groupwise differences by two-way ANOVA between control and LPS and *Double asterisk* indicates pairwise significance by Bonferroni’s post hoc

For glucose deprivation treatments, microglia were first washed thoroughly with fresh glucose-free DMEM/F-12 or 17.5 mM glucose DMEM/F-12 as appropriate and maintained with glucose-free or 17.5 mM glucose containing 1% FBS without antibiotics for 1 or 24 h prior to LPS treatment (100 ng/ml for 24 h in both 24- and 48-h treatments as outlined in Fig. 1a) and through the course of the experiment.

### Molecular Analyses

Nitric oxide levels were estimated by measuring the major metabolite nitrite using the Griess reaction [96, 97]. ELISAs for TNF and IL1β were performed according to the manufacturer’s instructions (R&D Systems, Minneapolis, MN).

All molecular analyses were normalized to the protein level of the cell lysate, measured using the BCA assay (Thermo-Fisher Pierce, Ottawa, ON) to account for any variability in cell numbers between wells.

MTT assay was performed by incubation with 0.5 mg/ml 3-(4,5-dimethylthiazol-2-yl)-2,5-diphenyltetrazolium bromide (MTT) for 30 min, lysis with dimethylsulfoxide and measurement of the oxidized formazan end-product at 540 nm. MTT results are presented uncorrected for total cell number but with cell counts determined in a parallel assay plate within the same experiment.

### Microscopy

Proliferation was assessed by incorporation of 5-ethynyl-2′-deoxyuridine (EdU) according to the manufacturer’s protocol (Click-IT EdU Imaging kit, Thermo-Fisher Scientific). Lipid droplets were visualized by staining with Bodipy 493/503 [98]. Phagocytosis was assayed by incubating treated cells with 1 × 10^7^ green fluorescent 1 μm carboxylate-modified latex beads (Sigma, Oakville, ON) for 2 h at 37 °C, 5% CO_2_, followed by thorough HBSS wash and fixation with 5% phosphate-buffered formalin as previously described [99]. Images were acquired using a Leica AF6000-LX microscope. Confocal microscopy was performed on a Leica TCS-SPE inverted microscope to validate bead internalization. Post-processing and quantitative analysis was performed using ImageJ using custom written macro functions as previously described [99]. Cell density measurements were determined by counting the total number of microglial cells in an automatically acquired 21-image array covering a total plate area of 25.5 mm^2^ for each condition.

### Statistical Analyses

Normality was determined using the D’Agostino-Pearson omnibus test. Overall significance was assessed using two-way ANOVA, with Bonferroni’s multiple comparison post-hoc analysis between groups. Pairwise comparisons were assessed using a Mann-Whitney *U* test. A *P* value of ≤0.05 was considered significant. Data are presented as the mean ± SEM. Each reported *N* indicates an independent experiment from a separate primary culture preparation.

## Results and Discussion

GD was carried out 1 h prior to LPS treatment for a total of 24 h to examine the acute effects of energy deprivation on the initiation of inflammatory functions [Fig. 1a]. GD of primary cultured microglia was insufficient to induce release of inflammatory modulators (TNF, IL1β or NO) but did significantly increase release of NO from reactive microglia after treatment with 100 ng/ml LPS [Fig. 1B] and showed a trend towards increased release of IL1β [Fig. 1c]. GD treatment was extended to 48 h in order to examine the possibility microglia were maintaining functions by depleting short-term stores of energy [Fig. 1a]. LPS treatment after 24 h of GD (48 h total timecourse) resulted in comparable trends towards increased release of NO, and a significant increase in the release of IL1β [Fig. 1b–c]. At both time points, TNF release was unaffected by glucose deprivation [Fig. 1d]. This observed increase of release suggests GD may result in a sensitization or ‘priming’ of microglial function to facilitate subsequent inflammatory release, but does not clarify the increased metabolic demand required to initiate inflammation. Cell metabolic activity, determined by the reduction of the tetrazolium dye 3-(4,5-dimethylthiazol-2-yl)-2,5-diphenyltetrazolium (MTT), was significantly decreased as a consequence of GD treatment in both the presence and absence of LPS treatment [Fig. 1e], and comparable effects were observed after 48 h of GD. As MTT reduction is dependent on NAD(P)H-dependent oxidoreductase enzymes, this suggests GD induced metabolic changes resulting in decreased oxidative phosphorylation and activity of the pentose phosphate pathway [100, 101]. Notably, these changes in MTT did not correlate with changes in cell numbers—parallel analysis of cell counts showed no significant difference in cell density as a consequence of glucose deprivation [Fig. 1e].

**Fig. 2:**
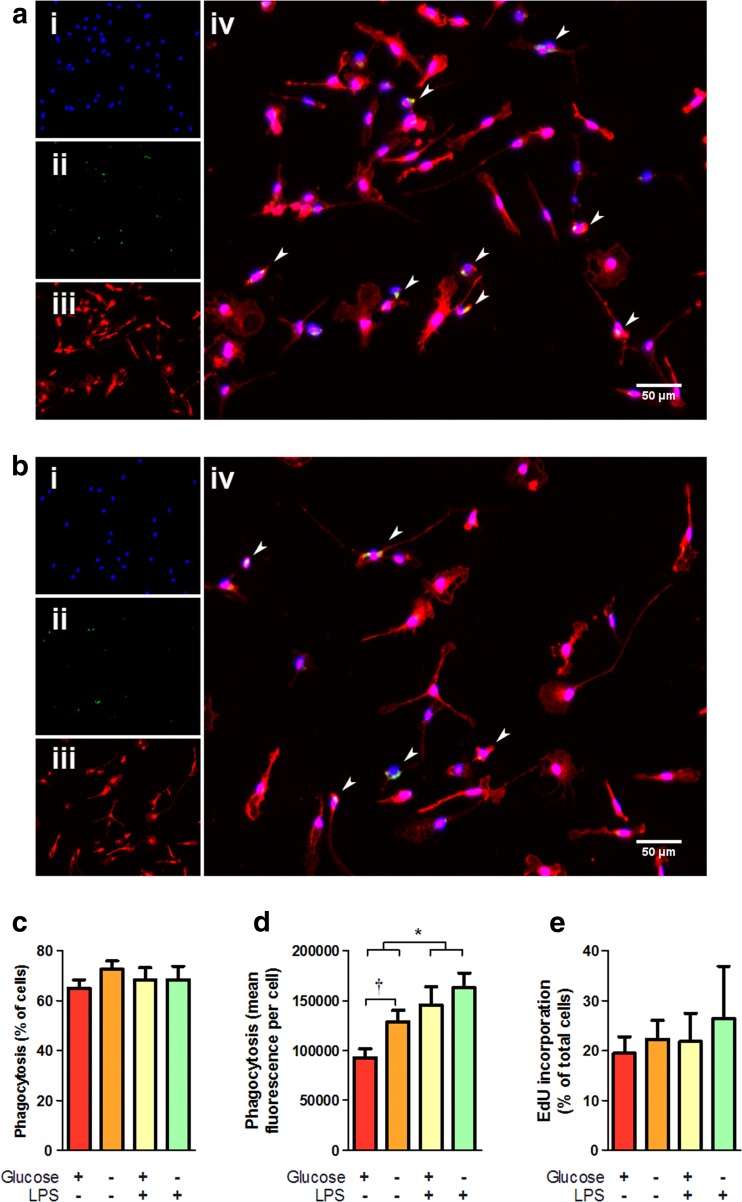
Glucose deprivation alters the function, but not the viability and proliferation, of microglia. Experiments were performed as in Fig. 1a, 24 h total time course. Immunofluorescence microscopy did not reveal any morphological differences between microglia cultured in normal glucose (**a**) or glucose deprived (**b**) media. Microglia were labelled with (*i*) Hoechst 33342, (*ii*) Bodipy 493/503, and (*iii*) Iba1, *arrowheads* in the overlay (*iv*) indicate microglia showing detectable lipid droplets as indicated by Bodipy 493/503 staining. **c** Phagocytosis as a portion of cells taking up fluorescent beads was not significantly affected by glucose deprivation (*N* = 6). **d** Phagocytic uptake, in terms of mean uptake of fluorescent beads per cell, was significantly increased with glucose deprivation (*N* = 6). **e** Proliferation, as measured by incorporation of the nucleotide analogue EdU, was not significantly affected by glucose deprivation (*N* = 3). *Asterisk* indicates groupwise differences between control and LPS treatment by two-way ANOVA, *Dagger* indicates pairwise difference between normal glucose and GD by Mann-Whitney *U* test

Cell morphology and culture confluence was not affected by GD treatment as determined by immunofluorescence microscopy [Fig. 2a–b]. Microglia labelled with the selective marker ionized Ca^2+^-binding adaptor protein-1 (Iba1) showed a variety of morphologies including a typical branched morphology suggestive of the surveillant state in both normal and glucose-deprived conditions. Notably, glucose-deprived cells showed a comparable confluence to control conditions and showed no indications of poor health (spherical morphology, condensed/fragmented nuclei, nor changes in media pH).

Phagocytic activity of primary microglia was assessed by measuring the uptake of fluorescent beads as previously described [99]. Neither glucose deprivation nor LPS treatment altered the proportion of cells involved in phagocytosis, remaining consistent between 65 and 72% in all conditions [Fig. 2c]; however, glucose deprivation significantly increased the uptake of beads. The mean fluorescence of phagocytic cells (mean fluorescence is proportional to the number of beads internalized per cell) was significantly higher in the GD condition relative to the normal glucose control in the absence of LPS treatment [Fig. 2d]. As expected, LPS increased the overall intake of beads significantly relative to the untreated condition, which abrogated the effect of glucose deprivation. This increased phagocytic activity precedes inflammatory activation, and is not accompanied by release of inflammatory mediators, but may reflect an increase in surveillance activities in response to metabolic stress. Coupled with the sensitization of inflammatory release observed in GD microglia, these data suggest GD may cue microglia to alter their function in anticipation of further insult. In contrast to the effects of glucose deprivation on inflammatory release and phagocytosis, GD did not affect either the viability of microglia (in terms of total cell numbers) or the proliferation rate in vitro. Proliferation, measured based on the incorporation of the nucleotide analogue 5-ethynyl-2′-deoxyuridine (EdU), was not significantly altered under any of the tested conditions, remaining consistent at ~20% [Fig. 2e].

**Fig. 3:**
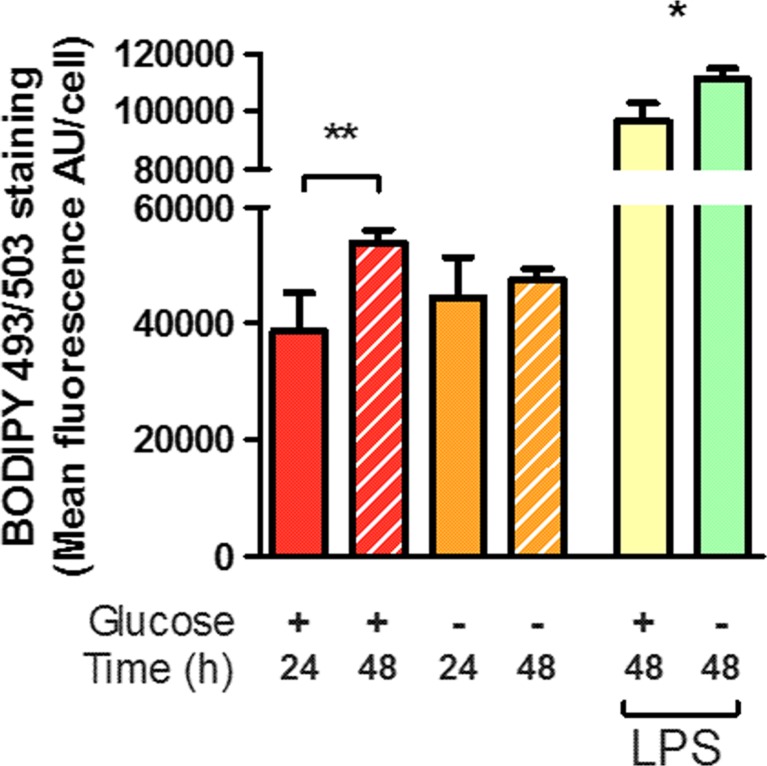
Glucose deprivation alters the accumulation of lipid droplets. Accumulation of lipid droplets was quantified by staining with BODIPY 493/503 and integration of green fluorescence within the soma of microglia identified by Hoechst 33342 and CD68 labelling, and is indicative of an increased size and/or number of lipid droplets. Fluorescence was significantly increased over the course of 48 h in the normal glucose condition, but not during glucose deprivation. Both normal and glucose-deprived cells showed significant increases in BODIPY 493/503 staining after LPS stimulation at either 24 or 48 h. *Asterisk* indicates significant groupwise differences by two-way ANOVA between control and LPS and *Double asterisk* indicates pairwise significance by Bonferroni’s post hoc (*N* = 4)

The survival, proliferation and functional capacity of microglia under glucose deprivation raises the question of alternative energy sources. In a number of cell types, nutritional deprivation results in mobilization of fatty acids from internal stores, including lipid droplets [102]. The accumulation of trigycerides in lipid droplets during nutritional deprivation is accompanied by an increase in β-oxidation of fatty acids as a survival mechanism, and been observed in a number of immortalized cell lines as well as rat primary astrocytes in vitro [98, 102, 103]. This accumulation of lipid droplets has been observed in in vivo models of ischemic stroke to correlate with CD11b + microglia/macrophages, and is suggested to contribute to a unique magnetic resonance signal observable in human patients after ischemic stroke using proton magnetic resonance spectroscopy [104]. To explore the influence of glucose treatment on lipid stores, accumulation of lipid droplets was assayed using the neutral lipid stain Bodipy 493/503. Immunofluorescence microscopy demonstrated Bodipy 493/503 staining was visible in a small subset of microglial cells after 24 h treatments [Fig. 2a–b] and lipid stores were predominantly associated with the cell soma and typically excluded from extended processes [Fig. 2a–b, Supplementary Fig. 1] and did not colocalize with the lysosomal marker CD68 [Supplemental Fig. 1]. Quantitative analysis of Bodipy 493/503 staining indicated lipid droplet accumulation was not significantly affected by 24 h GD; however, over the course of 48 h GD treatment, microglia did not significantly accumulate lipid stores [Fig. 3, Supplementary Fig. 1c]. This contrasts with the normal glucose condition in which a significant increase in lipid droplets was observed over 48 h. Both normal and GD microglia showed a significant increase in lipid droplets on challenge with LPS at 24 or 48 h [Fig. 3, Supplementary Fig. 1b, d], which suggests GD microglia are equally capable of reacting to external stimuli. The observed increase in lipid droplets size and/or number after exposure to LPS is consistent with observed LPS-induced accumulation of lipid droplets in the immortalized N9 microglial cell line [98, 103] and may be reflective of lipid redistribution as the cells undergo morphological changes from the branched, surveillant morphology to a spherical, reactive morphology rather than de novo synthesis of fatty acid stores. Alternately, as microglia are phagocytic cells and show significantly increased phagocytic uptake under conditions of glucose deprivation in the absence of LPS stimulus [Fig. 2d], this accumulation may reflect lipid taken in through phagocytosis of cellular debris. Notably, sustained LPS stimulation typically results in a high degree of cellular turnover as indicated by reduced cell density after LPS treatment [Fig. 1e] which may present opportunities for phagocytic uptake in the remaining cells. This pathway would provide continuous input to support vital functions of microglia during conditions of nutritional stress, including ischemia and would ultimately confound our assessment of lipid stores. Indeed, the observation that increased phagocytic activity during glucose deprivation was accompanied by a failure to further accumulate lipid stores may suggest stores are being actively depleted during the course of the experiment. In addition to the potential sustenance of vital function, lipid droplet accumulation in some cell types is associated with unfavourable outcomes: accumulation of lipid droplets in peripheral macrophages results from uptake of oxidized low-density lipoproteins and ultimately contributes to macrophage death and deposition in atherosclerotic plaques [105, 106], while comparable accumulation of lipid droplets in microglia/infiltrating macrophages during ischemic stroke contributes to the development of lipid-rich deposits in the core of injury [107]. It is as yet unclear if microglial accumulation of lipid droplets contributes to expression of either trophic or toxic behaviours in conditions of oxygen and/or glucose deprivation.

## Conclusion

The dynamic response of microglia to conditions of nutritional deprivation stands in contrast to our original hypothesis—we had expected a decrease in proliferation and a likely decrease in release of inflammatory mediators from reactive microglia. Glucose deprivation significantly affected cellular metabolism, as indicated by marked decrease in oxidative phosphorylation; however, rather than adopting a quiescent functional state, primary microglia increased phagocytic behaviours in the absence of glucose, and reactive microglia subject to GD increased the release of inflammatory mediators. This was notably accompanied by maintenance of the viability and proliferation of microglia. Thus, in the absence of glucose, microglia maintain or increase essential surveillance functions, and show sensitization of energy-intensive inflammatory processes. This unique aspect of microglial function stands in contrast to cells of the peripheral immune system, which have been shown to require oxidative phosphorylation and/or glycolysis for inflammatory functions—blockade of which results in a diminished inflammatory response [108–111] (and reviewed in [112]). While unexpected, these findings may reinforce the functional distinction between microglia and their counterparts in the peripheral immune system, and highlight specialized adaptations of inflammatory processes for diverse roles in the maintenance of brain homeostasis, development and plasticity [113]. It is not surprising that microglia are capable of utilizing alternate energy sources during glucose deprivation, as redundancy is a common feature of microglial function [114]. There remain unanswered questions of what primary energy stores are utilized by microglia during glucose deprivation, though the mobilization of fatty acids from internal stores would be expected given such observations in other cell types [102]. It is worth noting that microglia in the current study were not subjected to complete nutritional deprivation, but as glucose is the principal energy source in the brain it would be expected to be depleted rapidly during an ischemic or hypoglycemic event. The cell culture media used in the present study contain significant levels of amino acids which may be catabolized to intermediates capable of entry into the tricarboxylic acid cycle to sustain cellular metabolism, thus limiting the determination of a direct role of lipid stores. In the event of complete nutritional deprivation, we may expect a continuation of the observed trend towards sensitization, perhaps resulting in more direct activation of inflammatory processes due to the increased severity of the stressor. With complete nutritional deprivation, we may however observe a ceiling effect limiting the total time microglia are capable of surviving in the absence of external nutrients. A further caveat to the study presented is that cell culture media commonly contains up to 35 mM glucose, a level an order of magnitude higher than that typically seen in the CNS. Culture media in our normal glucose condition contained 17.5 mM glucose, which may still reflect a hyperglycemic condition; however, in our preliminary experiments, we did not observe any significant differences in inflammatory release between 17.5 and 1.75 mM glucose and chose to use the former to maintain consistency with the bulk of previously published in vitro studies.

The relevance of metabolic contributions to the pathology of mood disorders has gained interest in recent years, with several groups investigating bidirectional correlations between depression and diabetes or metabolic syndrome [115–117] and in particular correlations between circulating lipids (triglycerides, very low-, low-, and/or high-density lipoproteins) and suicidality [118, 119]. While these are observational studies of peripheral biomarkers, our data suggests metabolic states are capable of affecting microglia and as such may contribute to disease pathology through convergent effects on the inflammatory status of the CNS. As the key regulators of inflammation in the CNS, microglia possess a unique duality—acting as both a likely contributor to the pathophysiology of depressive disorders and as an ideal point of therapeutic intervention. The deficits present in our understanding of the trafficking and metabolism of lipids in microglia, and the potential consequences of dysregulation of lipid dynamics in microglia may represent an underexplored point of intervention to influence microglial fate and function in neurological, neurodegenerative and neuropsychiatric disorders.

## Electronic supplementary material

Supplementary Fig. 1 (DOCX 946 kb)

## References

[1] Heppner FL, Ransohoff RM, Becher B (2015). Immune attack: the role of inflammation in Alzheimer disease. Nat Rev Neurosci.

[2] Prokop S, Miller KR, Heppner FL (2013). Microglia actions in Alzheimer’s disease. Acta Neuropathol (Berl).

[3] Chao Y, Wong SC, Tan EK (2014). Evidence of inflammatory system involvement in Parkinson’s disease. Biomed Res Int.

[4] Deleidi M, Gasser T (2013). The role of inflammation in sporadic and familial Parkinson’s disease. Cell Mol Life Sci CMLS.

[5] Soulet D, Cicchetti F (2011). The role of immunity in Huntington’s disease. Mol Psychiatry.

[6] Ahmad M, Dar NJ, Bhat ZS (2014). Inflammation in ischemic stroke: mechanisms, consequences and possible drug targets. CNS Neurol Disord Drug Targets.

[7] Jin R, Liu L, Zhang S (2013). Role of inflammation and its mediators in acute ischemic stroke. J Cardiovasc Transl Res.

[8] Kim JY, Kawabori M, Yenari MA (2014). Innate inflammatory responses in stroke: mechanisms and potential therapeutic targets. Curr Med Chem.

[9] Kleinig TJ, Vink R (2009). Suppression of inflammation in ischemic and hemorrhagic stroke: therapeutic options. Curr Opin Neurol.

[10] Corps KN, Roth TL, McGavern DB (2015). Inflammation and neuroprotection in traumatic brain injury. JAMA Neurol.

[11] Helmy A, De Simoni M-G, Guilfoyle MR (2011). Cytokines and innate inflammation in the pathogenesis of human traumatic brain injury. Prog Neurobiol.

[12] Hinson HE, Rowell S, Schreiber M (2015). Clinical evidence of inflammation driving secondary brain injury: a systematic review. J Trauma Acute Care Surg.

[13] Zhou X, He X, Ren Y (2014). Function of microglia and macrophages in secondary damage after spinal cord injury. Neural Regen Res.

[14] Roy A, Campbell MK (2013). A unifying framework for depression: bridging the major biological and psychosocial theories through stress. Clin Investig Med Médecine Clin Exp.

[15] Schiepers OJG, Wichers MC, Maes M (2005). Cytokines and major depression. Prog Neuro-Psychopharmacol Biol Psychiatry.

[16] Zunszain PA, Hepgul N, Pariante CM (2013). Inflammation and depression. Curr Top Behav Neurosci.

[17] Feigenson KA, Kusnecov AW, Silverstein SM (2014). Inflammation and the two-hit hypothesis of schizophrenia. Neurosci Biobehav Rev.

[18] Frick LR, Williams K, Pittenger C (2013). Microglial dysregulation in psychiatric disease. Clin Dev Immunol.

[19] Najjar S, Pearlman DM (2014). Neuroinflammation and white matter pathology in schizophrenia: systematic review. Schizophr Res.

[20] Watanabe Y, Someya T, Nawa H (2010). Cytokine hypothesis of schizophrenia pathogenesis: evidence from human studies and animal models. Psychiatry Clin Neurosci.

[21] Meyer U, Feldon J, Dammann O (2011). Schizophrenia and autism: both shared and disorder-specific pathogenesis via perinatal inflammation?. Pediatr Res.

[22] Onore C, Careaga M, Ashwood P (2012). The role of immune dysfunction in the pathophysiology of autism. Brain Behav Immun.

[23] Rossignol DA, Frye RE (2014). Evidence linking oxidative stress, mitochondrial dysfunction, and inflammation in the brain of individuals with autism. Front Physiol.

[24] Theoharides TC, Asadi S, Patel AB (2013). Focal brain inflammation and autism. J Neuroinflammation.

[25] Baker DG, Nievergelt CM, O’Connor DT (2012). Biomarkers of PTSD: neuropeptides and immune signaling. Neuropharmacology.

[26] Wieck A, Grassi-Oliveira R, Hartmann do Prado C (2014). Neuroimmunoendocrine interactions in post-traumatic stress disorder: focus on long-term implications of childhood maltreatment. Neuroimmunomodulation.

[27] Rosenblat JD, Cha DS, Mansur RB, McIntyre RS (2014). Inflamed moods: a review of the interactions between inflammation and mood disorders. Prog Neuro-Psychopharmacol Biol Psychiatry.

[28] Slavich GM, Irwin MR (2014). From stress to inflammation and major depressive disorder: a social signal transduction theory of depression. Psychol Bull.

[29] Dowlati Y, Herrmann N, Swardfager W (2010). A meta-analysis of cytokines in major depression. Biol Psychiatry.

[30] Coulehan JL, Schulberg HC, Block MR (1990). Medical comorbidity of major depressive disorder in a primary medical practice. Arch Intern Med.

[31] Jiang M, Qin P, Yang X (2014). Comorbidity between depression and asthma via immune-inflammatory pathways: a meta-analysis. J Affect Disord.

[32] Krishnan KRR, Delong M, Kraemer H (2002). Comorbidity of depression with other medical diseases in the elderly. Biol Psychiatry.

[33] Oladeji BD, Gureje O (2013). The comorbidity between depression and diabetes. Curr Psychiatry Rep.

[34] Snyderman D, Wynn D (2009). Depression in cancer patients. Prim Care.

[35] Hanff TC, Furst SJ, Minor TR (2010). Biochemical and anatomical substrates of depression and sickness behavior. Isr J Psychiatry Relat Sci.

[36] Maes M, Berk M, Goehler L (2012). Depression and sickness behavior are Janus-faced responses to shared inflammatory pathways. BMC Med.

[37] Hauser P, Khosla J, Aurora H (2002). A prospective study of the incidence and open-label treatment of interferon-induced major depressive disorder in patients with hepatitis C. Mol Psychiatry.

[38] de Medeiros LPJ, Kayo M, Medeiros RBV (2014). Interferon-induced depression in patients with hepatitis C: an epidemiologic study. Rev Assoc Médica Bras.

[39] Smith KJ, Norris S, McKiernan S (2012). An exploration of depressive symptoms in hepatitis C patients taking interferon-alpha: increase in sickness behaviors but not negative cognitions. J Clin Exp Hepatol.

[40] Barakat R, Redzic Z (2015). The role of activated microglia and resident macrophages in the neurovascular unit during cerebral ischemia: is the jury still out?. Med Princ Pract Int J Kuwait Univ Health Sci Cent.

[41] Morrison HW, Filosa JA (2013). A quantitative spatiotemporal analysis of microglia morphology during ischemic stroke and reperfusion. J Neuroinflammation.

[42] Morioka T, Kalehua AN, Streit WJ (1993). Characterization of microglial reaction after middle cerebral artery occlusion in rat brain. J Comp Neurol.

[43] Zhang Z, Chopp M, Powers C (1997). Temporal profile of microglial response following transient (2 h) middle cerebral artery occlusion. Brain Res.

[44] Marks L, Carswell HV, Peters EE (2001). Characterization of the microglial response to cerebral ischemia in the stroke-prone spontaneously hypertensive rat. Hypertens Dallas Tex.

[45] Loubinoux I, Kronenberg G, Endres M (2012). Post-stroke depression: mechanisms, translation and therapy. J Cell Mol Med.

[46] Taylor WD, Steffens DC, Krishnan KR (2006). Psychiatric disease in the twenty-first century: the case for subcortical ischemic depression. Biol Psychiatry.

[47] Thomas AJ, O’Brien JT, Davis S (2002). Ischemic basis for deep white matter hyperintensities in major depression: a neuropathological study. Arch Gen Psychiatry.

[48] Chui H (2001). Dementia due to subcortical ischemic vascular disease. Clin Cornerstone.

[49] Lee MJ, Seo SW, Na DL (2014). Synergistic effects of ischemia and β-amyloid burden on cognitive decline in patients with subcortical vascular mild cognitive impairment. JAMA Psychiatry.

[50] Pluta R, Jolkkonen J, Cuzzocrea S (2011). Cognitive impairment with vascular impairment and degeneration. Curr Neurovasc Res.

[51] Villarreal AE, Barron R, Rao KS, Britton GB (2014). The effects of impaired cerebral circulation on Alzheimer’s disease pathology: evidence from animal studies. J Alzheimers Dis JAD.

[52] Kim HA, Miller AA, Drummond GR (2012). Vascular cognitive impairment and Alzheimer’s disease: role of cerebral hypoperfusion and oxidative stress. Naunyn Schmiedeberg's Arch Pharmacol.

[53] Pluta R, Furmaga-Jabłońska W, Maciejewski R (2013). Brain ischemia activates β- and γ-secretase cleavage of amyloid precursor protein: significance in sporadic Alzheimer’s disease. Mol Neurobiol.

[54] Pluta R, Jabłoński M, Ułamek-Kozioł M (2013). Sporadic Alzheimer’s disease begins as episodes of brain ischemia and ischemically dysregulated Alzheimer’s disease genes. Mol Neurobiol.

[55] McCrimmon RJ (2012). Update in the CNS response to hypoglycemia. J Clin Endocrinol Metab.

[56] Berge LI, Riise T (2015). Comorbidity between type 2 diabetes and depression in the adult population: directions of the association and its possible pathophysiological mechanisms. Int J Endocrinol.

[57] Martinac M, Pehar D, Karlović D (2014). Metabolic syndrome, activity of the hypothalamic-pituitary-adrenal axis and inflammatory mediators in depressive disorder. Acta Clin Croat.

[58] Emanuele E, Martinelli V, Carlin MV (2011). Serum levels of soluble receptor for advanced glycation endproducts (sRAGE) in patients with different psychiatric disorders. Neurosci Lett.

[59] Spauwen PJJ, van Eupen MGA, Köhler S (2015). Associations of advanced glycation end-products with cognitive functions in individuals with and without type 2 diabetes: the Maastricht Study. J Clin Endocrinol Metab.

[60] Musen G, Lyoo IK, Sparks CR (2006). Effects of type 1 diabetes on gray matter density as measured by voxel-based morphometry. Diabetes.

[61] Tambuyzer BR, Ponsaerts P, Nouwen EJ (2009). Microglia: gatekeepers of central nervous system immunology. J Leukoc Biol.

[62] Katsumoto A, Lu H, Miranda AS, Ransohoff RM (2014). Ontogeny and functions of central nervous system macrophages. J Immunol Baltim Md.

[63] Prinz M, Priller J (2014). Microglia and brain macrophages in the molecular age: from origin to neuropsychiatric disease. Nat Rev Neurosci.

[64] Prinz M, Tay TL, Wolf Y, Jung S (2014). Microglia: unique and common features with other tissue macrophages. Acta Neuropathol (Berl).

[65] Schwartz M, Butovsky O, Brück W, Hanisch U-K (2006). Microglial phenotype: is the commitment reversible?. Trends Neurosci.

[66] Boche D, Perry VH, Nicoll JAR (2013). Review: activation patterns of microglia and their identification in the human brain. Neuropathol Appl Neurobiol.

[67] Town T, Nikolic V, Tan J (2005). The microglial “activation” continuum: from innate to adaptive responses. J Neuroinflammation.

[68] Raivich G (2005). Like cops on the beat: the active role of resting microglia. Trends Neurosci.

[69] Sierra A, Beccari S, Diaz-Aparicio I (2014). Surveillance, phagocytosis, and inflammation: how never-resting microglia influence adult hippocampal neurogenesis. Neural Plast.

[70] Tremblay M-È (2011). The role of microglia at synapses in the healthy CNS: novel insights from recent imaging studies. Neuron Glia Biol.

[71] Zanier ER, Fumagalli S, Perego C (2015). Shape descriptors of the “never resting” microglia in three different acute brain injury models in mice. Intensive Care Med Exp.

[72] Kroner A, Greenhalgh AD, Zarruk JG (2014). TNF and increased intracellular iron alter macrophage polarization to a detrimental M1 phenotype in the injured spinal cord. Neuron.

[73] Morsch M, Radford R, Lee A (2015). In vivo characterization of microglial engulfment of dying neurons in the zebrafish spinal cord. Front Cell Neurosci.

[74] Schafer DP, Lehrman EK, Kautzman AG (2012). Microglia sculpt postnatal neural circuits in an activity and complement-dependent manner. Neuron.

[75] Sierra A, Encinas JM, Deudero JJP (2010). Microglia shape adult hippocampal neurogenesis through apoptosis-coupled phagocytosis. Cell Stem Cell.

[76] Girard S, Brough D, Lopez-Castejon G (2013). Microglia and macrophages differentially modulate cell death after brain injury caused by oxygen-glucose deprivation in organotypic brain slices. Glia.

[77] Ajami B, Bennett JL, Krieger C (2007). Local self-renewal can sustain CNS microglia maintenance and function throughout adult life. Nat Neurosci.

[78] Ajami B, Bennett JL, Krieger C (2011). Infiltrating monocytes trigger EAE progression, but do not contribute to the resident microglia pool. Nat Neurosci.

[79] Barakat R, Redzic Z (2015). Differential cytokine expression by brain microglia/macrophages in primary culture after oxygen glucose deprivation and their protective effects on astrocytes during anoxia. Fluids Barriers CNS.

[80] Eyo U, Dailey ME (2012). Effects of oxygen-glucose deprivation on microglial mobility and viability in developing mouse hippocampal tissues. Glia.

[81] Hall AA, Leonardo CC, Collier LA (2009). Delayed treatments for stroke influence neuronal death in rat organotypic slice cultures subjected to oxygen glucose deprivation. Neuroscience.

[82] Bell MT, Puskas F, Agoston VA (2013). Toll-like receptor 4-dependent microglial activation mediates spinal cord ischemia-reperfusion injury. Circulation.

[83] Lalancette-Hébert M, Gowing G, Simard A (2007). Selective ablation of proliferating microglial cells exacerbates ischemic injury in the brain. J Neurosci.

[84] Montero M, González B, Zimmer J (2009). Immunotoxic depletion of microglia in mouse hippocampal slice cultures enhances ischemia-like neurodegeneration. Brain Res.

[85] Chen W, Ostrowski RP, Obenaus A, Zhang JH (2009). Prodeath or prosurvival: two facets of hypoxia inducible factor-1 in perinatal brain injury. Exp Neurol.

[86] Jantzie LL, Cheung P-Y, Todd KG (2005). Doxycycline reduces cleaved caspase-3 and microglial activation in an animal model of neonatal hypoxia-ischemia. J Cereb Blood Flow Metab Off J Int Soc Cereb Blood Flow Metab.

[87] Lai AY, Todd KG (2006). Hypoxia-activated microglial mediators of neuronal survival are differentially regulated by tetracyclines. Glia.

[88] Shay JES, Celeste Simon M (2012). Hypoxia-inducible factors: crosstalk between inflammation and metabolism. Semin Cell Dev Biol.

[89] Choi SJ, Shin IJ, Je K-H (2013). Hypoxia antagonizes glucose deprivation on interleukin 6 expression in an Akt dependent, but HIF-1/2α independent manner. PLoS One.

[90] Eyo UB, Miner SA, Ahlers KE (2013). P2X7 receptor activation regulates microglial cell death during oxygen-glucose deprivation. Neuropharmacology.

[91] Ziemka-Nałęcz M, Stanaszek L, Zalewska T (2013). Oxygen-glucose deprivation promotes gliogenesis and microglia activation in organotypic hippocampal slice culture: involvement of metalloproteinases. Acta Neurobiol Exp (Warsz).

[92] Gimeno-Bayón J, López-López A, Rodríguez MJ, Mahy N (2014). Glucose pathways adaptation supports acquisition of activated microglia phenotype. J Neurosci Res.

[93] Uhlemann R, Gertz K, Boehmerle W (2015). Actin dynamics shape microglia effector functions. Brain Struct Funct.

[94] Orihuela R, McPherson CA, Harry GJ (2016). Microglial M1/M2 polarization and metabolic states. Br J Pharmacol.

[95] Saura J, Tusell JM, Serratosa J (2003). High-yield isolation of murine microglia by mild trypsinization. Glia.

[96] Tsikas D (2007). Analysis of nitrite and nitrate in biological fluids by assays based on the Griess reaction: appraisal of the Griess reaction in the L-arginine/nitric oxide area of research. J Chromatogr B Analyt Technol Biomed Life Sci.

[97] Griess P (1879). Bemerkungen zu der Abhandlung der HH. Weselsky und Benedikt ,Ueber einige Azoverbindungen. Berichte Dtsch Chem Ges.

[98] Khatchadourian A, Bourque SD, Richard VR (2012). Dynamics and regulation of lipid droplet formation in lipopolysaccharide (LPS)-stimulated microglia. Biochim Biophys Acta.

[99] Churchward MA, Todd KG (2014). Statin treatment affects cytokine release and phagocytic activity in primary cultured microglia through two separable mechanisms. Mol Brain.

[100] Berridge MV, Tan AS (1993). Characterization of the cellular reduction of 3-(4,5-dimethylthiazol-2-yl)-2,5-diphenyltetrazolium bromide (MTT): subcellular localization, substrate dependence, and involvement of mitochondrial electron transport in MTT reduction. Arch Biochem Biophys.

[101] Berridge MV, Herst PM, Tan AS (2005). Tetrazolium dyes as tools in cell biology: new insights into their cellular reduction. Biotechnol Annu Rev.

[102] Cabodevilla AG, Sánchez-Caballero L, Nintou E (2013). Cell survival during complete nutrient deprivation depends on lipid droplet-fueled β-oxidation of fatty acids. J Biol Chem.

[103] Tremblay M-E, Zhang I, Bisht K (2016). Remodeling of lipid bodies by docosahexaenoic acid in activated microglial cells. J Neuroinflammation.

[104] Gasparovic C, Rosenberg GA, Wallace JA (2001). Magnetic resonance lipid signals in rat brain after experimental stroke correlate with neutral lipid accumulation. Neurosci Lett.

[105] Adibhatla RM, Hatcher JF (2010). Lipid oxidation and peroxidation in CNS health and disease: from molecular mechanisms to therapeutic opportunities. Antioxid Redox Signal.

[106] Yuan Y, Li P, Ye J (2012). Lipid homeostasis and the formation of macrophage-derived foam cells in atherosclerosis. Protein Cell.

[107] Saunders DE, Howe FA, van den Boogaart A (1997). Discrimination of metabolite from lipid and macromolecule resonances in cerebral infarction in humans using short echo proton spectroscopy. J Magn Reson Imaging JMRI.

[108] Infantino V, Convertini P, Cucci L (2011). The mitochondrial citrate carrier: a new player in inflammation. Biochem J.

[109] Mills EL, Kelly B, Logan A (2016). Succinate dehydrogenase supports metabolic repurposing of mitochondria to drive inflammatory macrophages. Cell.

[110] Krawczyk CM, Holowka T, Sun J (2010). Toll-like receptor-induced changes in glycolytic metabolism regulate dendritic cell activation. Blood.

[111] Chang C-H, Curtis JD, Maggi LB (2013). Posttranscriptional control of T cell effector function by aerobic glycolysis. Cell.

[112] O’Neill LAJ, Kishton RJ, Rathmell J (2016). A guide to immunometabolism for immunologists. Nat Rev Immunol.

[113] Crotti A, Ransohoff RM (2016). Microglial physiology and pathophysiology: insights from genome-wide transcriptional profiling. Immunity.

[114] Casano AM, Peri F (2015). Microglia: multitasking specialists of the brain. Dev Cell.

[115] Koponen H, Kautiainen H, Leppänen E (2015). Association between suicidal behaviour and impaired glucose metabolism in depressive disorders. BMC Psychiatry.

[116] Mansur RB, Brietzke E, McIntyre RS (2015). Is there a “metabolic-mood syndrome”? A review of the relationship between obesity and mood disorders. Neurosci Biobehav Rev.

[117] Pan A, Keum N, Okereke OI (2012). Bidirectional association between depression and metabolic syndrome: a systematic review and meta-analysis of epidemiological studies. Diabetes Care.

[118] Baek JH, Kang E-S, Fava M (2014). Serum lipids, recent suicide attempt and recent suicide status in patients with major depressive disorder. Prog Neuro-Psychopharmacol Biol Psychiatry.

[119] da Graça CM, Nardin P, Buffon A (2014). Serum triglycerides, but not cholesterol or leptin, are decreased in suicide attempters with mood disorders. J Affect Disord.

