# Supplementary material for: Microglial Function during Glucose Deprivation: Inflammatory and Neuropsychiatric Implications
Source: Mol Neurobiol. 2017 Feb 7;55(2):1477–87. doi: 10.1007/s12035-017-0422-9 (PMC5820372; doi:10.1007/s12035-017-0422-9)
Supplement: Supplementary file 1 — (DOCX 946 kb) [file 12035_2017_422_MOESM1_ESM.docx]

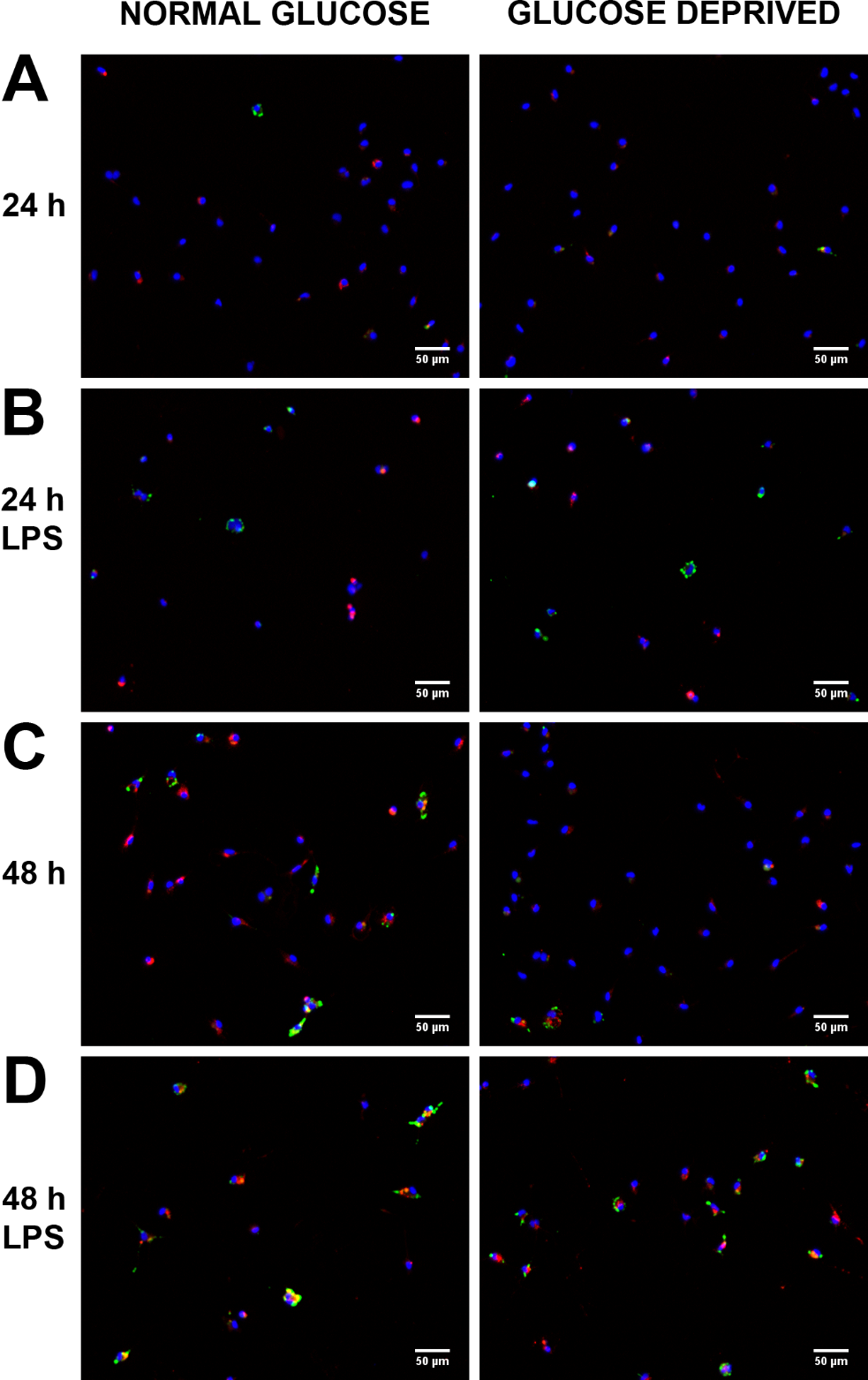
Supplementary Figure 1: Lipid droplet imaging in cultured microglia. Microglia were labelled with Hoechst 33342 (blue), Bodipy 493/503 (green), and immunolabelled for the microglia-specific / lysosomal marker CD68 (red). Microglia were cultured with or without glucose for (A) 24 hours, (B) 24 hours with LPS treatment, (C) 48 hours, or (D) 48 hours with 24 hour LPS treatment.
